# Peripheral T-cell transitions following proton pump inhibitor therapy in eosinophilic esophagitis

**DOI:** 10.1093/cei/uxag051

**Published:** 2026-08-20

**Authors:** John Plate, Christine Lingblom, Helen Larsson

**Affiliations:** Department of Otorhinolaryngology Head and Neck Surgery, Region Västra Götaland, NU-Hospital Group, Trollhättan, Sweden; Department of Otorhinolaryngology, Head and Neck Surgery, Institute of Clinical Sciences, Sahlgrenska Academy, University of Gothenburg, Gothenburg, Sweden; Department of Infectious Diseases, Institute of Biomedicine, The Sahlgrenska Academy, University of Gothenburg, Gothenburg, Sweden; Department of Clinical Microbiology, Sahlgrenska University Hospital, Gothenburg, Sweden; Department of Otorhinolaryngology Head and Neck Surgery, Region Västra Götaland, NU-Hospital Group, Trollhättan, Sweden; Department of Otorhinolaryngology, Head and Neck Surgery, Institute of Clinical Sciences, Sahlgrenska Academy, University of Gothenburg, Gothenburg, Sweden

**Keywords:** eosinophilic esophagitis, cluster analysis, CyTOF, T cells, human, proton pump inhibitors

## Abstract

Eosinophilic esophagitis (EoE) is a chronic allergic inflammatory condition characterized by eosinophilic infiltration of the esophagus and dysregulated T-cell responses. Proton pump inhibitors (PPIs) are a treatment option for patients with EoE; however, biomarkers for noninvasive monitoring of treatment response are lacking. In this study, we prospectively enrolled 20 patients with EoE who were treated with PPIs, of whom 19 completed their treatment, and analyzed peripheral blood immune profiles using mass cytometry (CyTOF), comparing them with age- and sex-matched healthy controls. Histologically, 14 of 19 patients achieved histologic remission, whereas immunophenotyping was performed in 17 patients (12 responders and 5 nonresponders) because 2 samples contained insufficient cell numbers. The patients were also evaluated using the EoE Histology Scoring System (EoE-HSS; stage and grade), endoscopic findings, and validated questionnaires. Unsupervised clustering identified key T-cell populations altered by treatment, including Th2 central memory CD4^+^ T cells and CD8^+^ terminal effector and naïve subsets. Successful PPI therapy was associated with expansion of FOXP3^+^ T cells and reduction in cytotoxic CD56^+^ CD8^+^ T cells, modulations not detected in nonresponders. Notably, peripheral CD4^+^ T-cell counts correlated with histologic disease severity (EoE-HSS), whereas eosinophil counts did not. These findings highlight dynamic shifts in circulating T-cell phenotypes following PPI therapy and suggest that expansion of FOXP3^+^ T-cells and reduction of CD56^+^ CD8^+^ T-cell populations may represent candidate treatment-associated immune signatures in EoE. However, given the exploratory nature of this high-dimensional immunophenotyping study and the limited sample size, these findings should be considered hypothesis-generating and require validation in larger independent cohorts.

## Teaser

Eosinophilic esophagitis (EoE) is a chronic allergic inflammatory condition characterized by eosinophilic infiltration of the esophagus and dysregulated T-cell responses. Proton pump inhibitors (PPIs) are a treatment option for patients with EoE, however, biomarkers for non-invasive monitoring of treatment response are lacking. In this study, we prospectively enrolled twenty patients with EoE who were treated with PPIs, of whom nineteen completed their treatment, and analyzed peripheral blood immune profiles using mass cytometry (CyTOF), comparing them with age- and sex-matched healthy controls.

## Introduction

Eosinophilic esophagitis (EoE) is a chronic, antigen-driven inflammatory disease of the esophagus characterized by a type 2 immune response, predominantly triggered by food or inhalant allergens [1, 2]. Dysregulated immune activity leads to sustained eosinophilic infiltration of esophageal mucosa, which is normally devoid of eosinophils in healthy individuals [3, 4]. While eosinophils represent the hallmark effector cells implicated in tissue damage, a complex cellular network including increased numbers of T lymphocytes, basophils, mast cells, and B cells also contributes to disease pathogenesis and the perpetuation of inflammation [1, 2, 5, 6].

The T-cell compartment in EoE is of particular interest, as Th2 (T helper 2) cytokines such as interleukin (IL)-4, IL-5, and IL-13 orchestrate the recruitment and activation of eosinophils and other inflammatory cells [7, 8]. In addition to classical Th2 CD4^+^ T cells, recent studies have identified alterations in regulatory T-cell populations and cytotoxic CD8^+^ T cells in both blood and esophageal tissue, highlighting their potential roles in disease progression and response to therapy [9, 10]. Moreover, cytotoxic CD8^+^ T cells have been linked to epithelial cell injury and fibrosis [10], emphasizing the multifaceted involvement of T cells beyond eosinophilic inflammation.

Clinically, EoE predominantly presents in adults as dysphagia and food impaction, symptoms that profoundly impact quality of life and may lead to complications including fibrotic esophageal remodeling, stricture formation, and esophageal rupture if left untreated [11–14]. Current therapeutic strategies include dietary allergen elimination [15], swallowed topical corticosteroids, proton pump inhibitors (PPIs) [16], and, more recently, biologic therapies such as dupilumab, an anti-IL-4Rα monoclonal antibody that blocks IL-4 and IL-13 signaling pathways, key drivers of type 2 inflammation [17].

At present, treatment efficacy is evaluated through histologic examination of esophageal mucosa, with remission defined as fewer than 15 eosinophils per high-power field (HPF) and deep remission as fewer than 5 eosinophils/HPF. However, eosinophil counts alone provide limited insight into the broader inflammatory milieu and may be affected by the patchy distribution of eosinophils in the mucosa [18]. The EoE Histology Scoring System (EoE-HSS) was developed to provide a more comprehensive histologic assessment by incorporating additional parameters such as basal zone hyperplasia, dilated intercellular spaces, and lamina propria fibrosis, thereby better reflecting tissue remodeling and disease activity [19]. Complementing histology, endoscopic evaluation using the Eosinophilic Esophagitis Endoscopic Reference Score (EREFS) quantifies endoscopic features including edema, rings, exudates, furrows, and strictures, providing a validated and reproducible method to assess both inflammation and structural remodeling [20]. However, inflammatory burden cannot be fully captured by EREFS alone [21].

Despite advances in diagnostic modalities, repeated endoscopy with biopsy remains the clinical gold standard for monitoring treatment response. This approach is invasive, costly, and burdensome for patients, underscoring the need for reliable noninvasive biomarkers to monitor disease activity [22]. Peripheral blood immune profiling represents a promising strategy, as it enables repeated, minimally invasive assessment of systemic immune alterations that may reflect mucosal inflammation.

In this study, our primary aim was to determine whether blood-based immune markers, particularly T-cell phenotypes, are associated with treatment response and disease activity in EoE. To address this, we developed a high-dimensional mass cytometry (CyTOF) panel targeting major immune populations with a focus on T-cell subsets, including memory, regulatory, and cytotoxic phenotypes. Using unsupervised clustering (FlowSOM) and dimensionality reduction (opt-SNE), we compared immune profiles before and after PPI therapy, using age- and sex-matched healthy controls for comparison.

A secondary objective was to assess the clinical efficacy of PPIs using histologic (eosinophils/HPF, EoE-HSS), endoscopic (EREFS), and the symptom-based Eosinophilic Esophagitis Activity Index (EEsAI) and Watson Dysphagia Scale (WDS) assessments, and to explore correlations between these clinical outcomes and peripheral T-cell immune markers. We hypothesized that successful PPI therapy would be accompanied by distinct shifts in circulating T-cell populations, which may identify candidate immune signatures associated with treatment response.

## Methods and materials

### Study design

Twenty adult patients (aged ≥18 years) with active EoE, defined as having >15 eosinophils per HPF and symptoms of esophageal dysfunction, were prospectively recruited at Northern Älvsborg County Hospital, Trollhattan, Sweden. Patients were enrolled regardless of their anticipated response to treatment.

**Figure 1:**
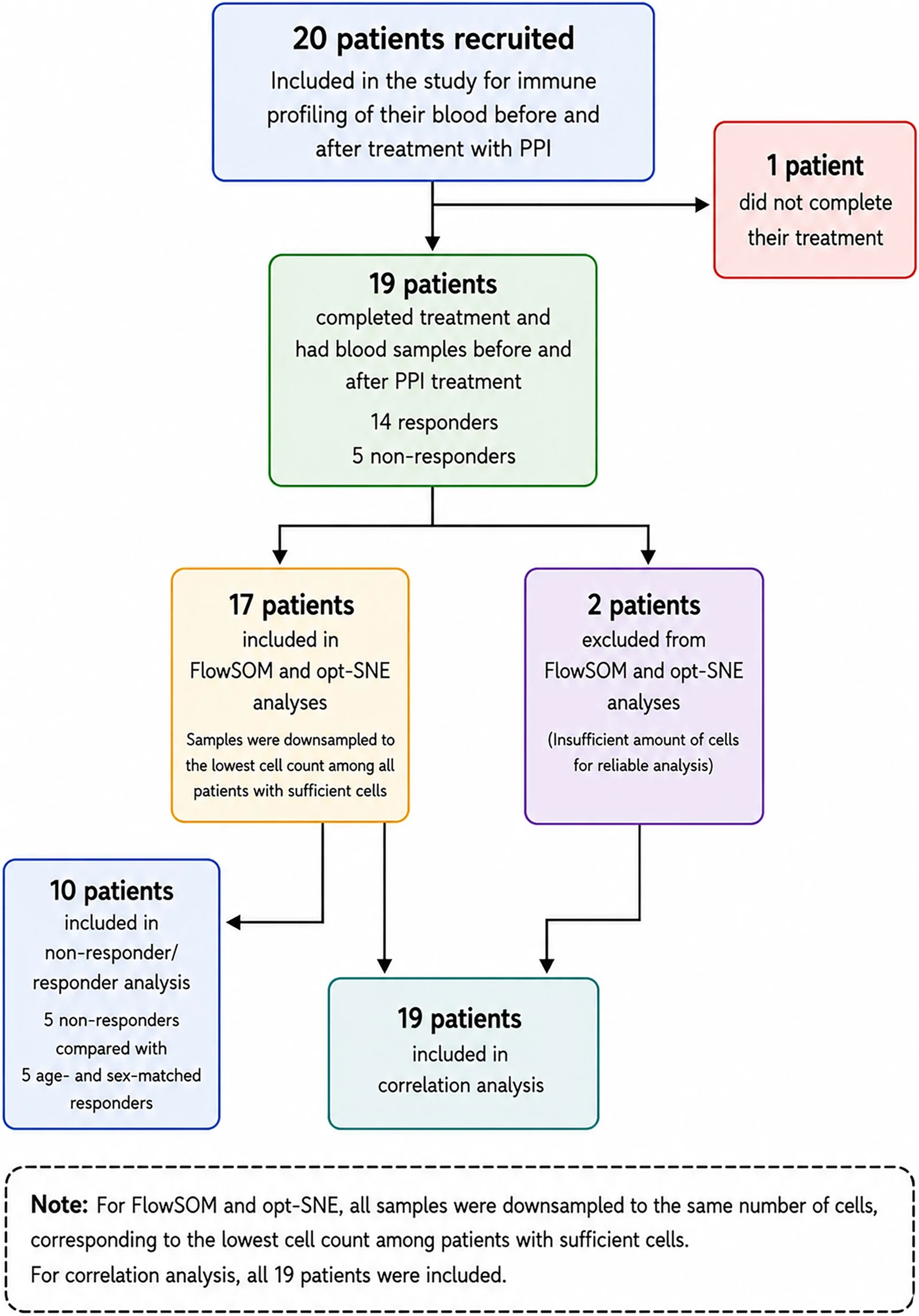
Flowchart of patient inclusion. Twenty patients were enrolled, of whom 19 completed PPI treatment. In the full clinical cohort, 14 of 19 patients achieved histologic remission (<15 eosinophils/HPF), whereas 5 were classified as nonresponders. Two responder samples contained insufficient cell numbers for CyTOF analysis and were therefore excluded from immunophenotyping. Consequently, the CyTOF cohort comprised 17 patients (12 responders and 5 nonresponders).

All participants received a 12-week course of PPI therapy (omeprazole, 20 mg, twice daily). No patients received additional therapies besides PPI treatment during the study period. Specifically, none of the patients were treated with swallowed topical corticosteroids, dupilumab, or allergen elimination diets, with the exception of one patient with celiac disease who was already on a gluten-free diet prior to inclusion in the study. Concomitant therapies may influence peripheral blood immune cell profiles. Blood samples were collected before and after the treatment period. For comparison, 20 age- and sex-matched healthy controls without esophageal symptoms were also recruited. Exclusion criteria for EoE patients included ongoing immunomodulatory therapy or failure to follow the treatment protocol. A flow diagram of all patients can be seen in Figure 1.

The study was approved by the Regional Ethical Review Board in Gothenburg, Sweden. Written informed consent was obtained from all participants. All procedures were conducted in adherence to local ethical standards and in accordance with the 1964 Helsinki Declaration.

### Patient outcomes

COREOS-recommended guidelines [23] was followed to evaluate treatment response, incorporating standardized clinical, histological, and endoscopic assessments. These included:

- Peak eosinophil count (remission <15 eosinophils/HPF)
- The EoE-HSS, where a score of 0 indicates minimal histologic inflammation and 1 indicates high levels of inflammation [19].
- The EREFS, applied using a modified scoring system as recommended by the COREOS group. This version has a maximum score of 8 points, where 0 represents minimal endoscopic signs of inflammation and 8 represents severe inflammation [20, 23].
- To assess patient-reported outcomes, two EoE-specific, Swedish-validated questionnaires were used:
- The EEsAI, ranging from 0 (no symptom burden) to 100 (high symptom burden) [24].
- The WDS, ranging from 0 (no dysphagia) to 45 (severe dysphagia) [25].

Demographic and clinical characteristics are summarized in Supplementary Table S1, and average scoring results for the EoE patients are presented in Supplementary Table S2.

### Mass cytometry

Heparinized venous blood samples were processed for CyTOF analysis. Erythrocytes were lysed using ammonium chloride lysis buffer for 15 minutes at room temperature (RT), after which the remaining leukocytes were washed with Maxpar PBS (Fluidigm, South San Francisco, CA). Heparinized blood was used to minimize nonspecific binding between cationic proteins in eosinophils and metal isotopes [26]. Cell suspensions were incubated with Cell-ID Cisplatin (5 µM, Fluidigm) for 5 minutes at RT to assess cell viability, followed by a wash step. Cells were then incubated with Human TruStain FcX (BioLegend, San Diego, CA) to block Fc receptors, together with a surface antibody cocktail (see Supplementary Table S3), for 30 minutes at RT. Following staining, cells were washed and fixed with 1.6% formaldehyde for 10 minutes at RT, then permeabilized using the Foxp3/Transcription Factor Staining Buffer Set (eBioscience, San Diego, CA) for 1 hour at RT. Intracellular staining was then performed using a second antibody cocktail (Supplementary Table S3) for 1.5 hours at RT. After staining, cells were washed and incubated with an intercalation solution containing 62.5 nM Cell-ID Intercalator-Ir (125 µM) in Maxpar Fix and Perm Buffer (Fluidigm) for 45 minutes at RT. Samples were then resuspended in Maxpar PBS and stored overnight at 4°C. Prior to acquisition, cells were diluted in MilliQ H_2_O to a concentration of 1 × 10^6^ cells/ml, and 0.1× EQ Four Element Calibration Beads (Fluidigm) were added. Data acquisition was performed using a Helios CyTOF instrument with CyTOF Software v7.0 (Fluidigm). Gating and data analysis were conducted using FlowJo software v10.8.0 (Tree Star Inc., Ashland, OR) (see Supplementary Figure S1). Results are presented as the percentage of cells expressing each marker.

### High-dimensional data analysis and statistics

CyTOF data (n = 34) were pregated for T-cell populations in FlowJo (FlowJo, LLC, BD) prior to export into Cytobank (Beckman Coulter, Brea, CA) for unsupervised analyses. Sample-specific gating was used to account for inter-individual variation and optimize population identification across samples. Files were imported to Cytobank where FlowSOM [27] was used to define clusters, which were visualized using metacluster box plots. FlowSOM clustering was performed using the markers listed in Supplementary Table S3. Cluster identities were subsequently assigned based on relative marker expression patterns and established immunophenotypic conventions. The analysis was followed by Opt-SNE (optimized t-distributed stochastic neighbor embedding) [28] for dimensionality reduction to visualize phenotypic relationships between the defined clusters in a scatterplot map. Opt-SNE is an improved version of t-SNE [29]. The purpose of opt-SNE is to automatically optimize t-SNE parameters, improve reproducibility, better preserve population structure, and handle very large datasets more efficiently. Cell populations were annotated based on relative marker expression profiles following FlowSOM clustering and opt-SNE visualization. Population identities represent phenotypic interpretations based on established immunophenotypic conventions rather than direct functional characterization. Given the exploratory nature of this study and the limited sample size, correction for multiple testing was performed, and all statistical analyses should be interpreted as hypothesis-generating. Exact *P*-values, adjusted *P*-values using the Benjamini–Hochberg false discovery rate (FDR) procedure, estimated effect sizes using Hedges’ *g*, and 95% confidence intervals (95% CIs) for mean differences calculated using the Welch two-sample method were reported. The FDR correction was performed across 37 populations, as these variables formed the basis of the high-dimensional clustering approach. In addition, Bonferroni-adjusted *P*-values were calculated (Supplementary Table S4). Given the limited sample size when comparing non-responders to responders, correction for multiple testing was not performed. Univariate statistical analyses of molecular marker expression and specific cell subpopulations were performed using GraphPad Prism (version 9.2.0; GraphPad, San Diego, CA). Correlations between datasets were assessed using Spearman’s rank correlation coefficient. A *P*-value of <0.05 was considered statistically significant.

## Results

### Proton pump inhibitors successfully treat approximately 70% of patients with active eosinophilic esophagitis

Of the 20 patients recruited, nineteen were included in the study, 1 was excluded due to noncompliance with treatment. Based on histologic findings, PPIs proved to be an effective therapy. Of the 19 patients who completed treatment, 14 (74%, 14/19) achieved histologic remission (<15 eos/HPF). Two patients with histologic remission had insufficient cell numbers for CyTOF analysis and were therefore excluded from the immunophenotyping analyses. Consequently, the CyTOF cohort comprised 17 patients, including 12 responders and 5 nonresponders. In the responder group, eosinophil counts (eosinophils/HPF) decreased significantly from a median of 45 (range 16–122) before treatment to 13 (range 0–83) after treatment. Correspondingly, both the EoE-HSS stage and grade showed significant reductions. The EoE-HSS stage decreased from 0.82 (range 0.62–0.96) to 0.48 (range 0–0.86), and the EoE-HSS grade decreased from 0.64 (range 0.33–0.92) to 0.33 (range 0–0.67). Endoscopic scoring (EREFS) improved from 3.7 (range 0–7) to 2.0 (range 0–4) after treatment. Similarly, symptom-related scores declined: EEsAI decreased from 43.4 (range 0–74) to 24 (range 0–63), and the WDS decreased from 16.1 (range 4.5–25.5) to 11.3 (range 0–23). Two patients completed treatment but were excluded from the FlowSOM and opt-SNE analyses because insufficient cell numbers precluded reliable clustering analysis. Consequently, 17 patients were included in the immunological analyses.

### Changes in T-cell populations following proton pump inhibitor therapy

**Figure 2:**
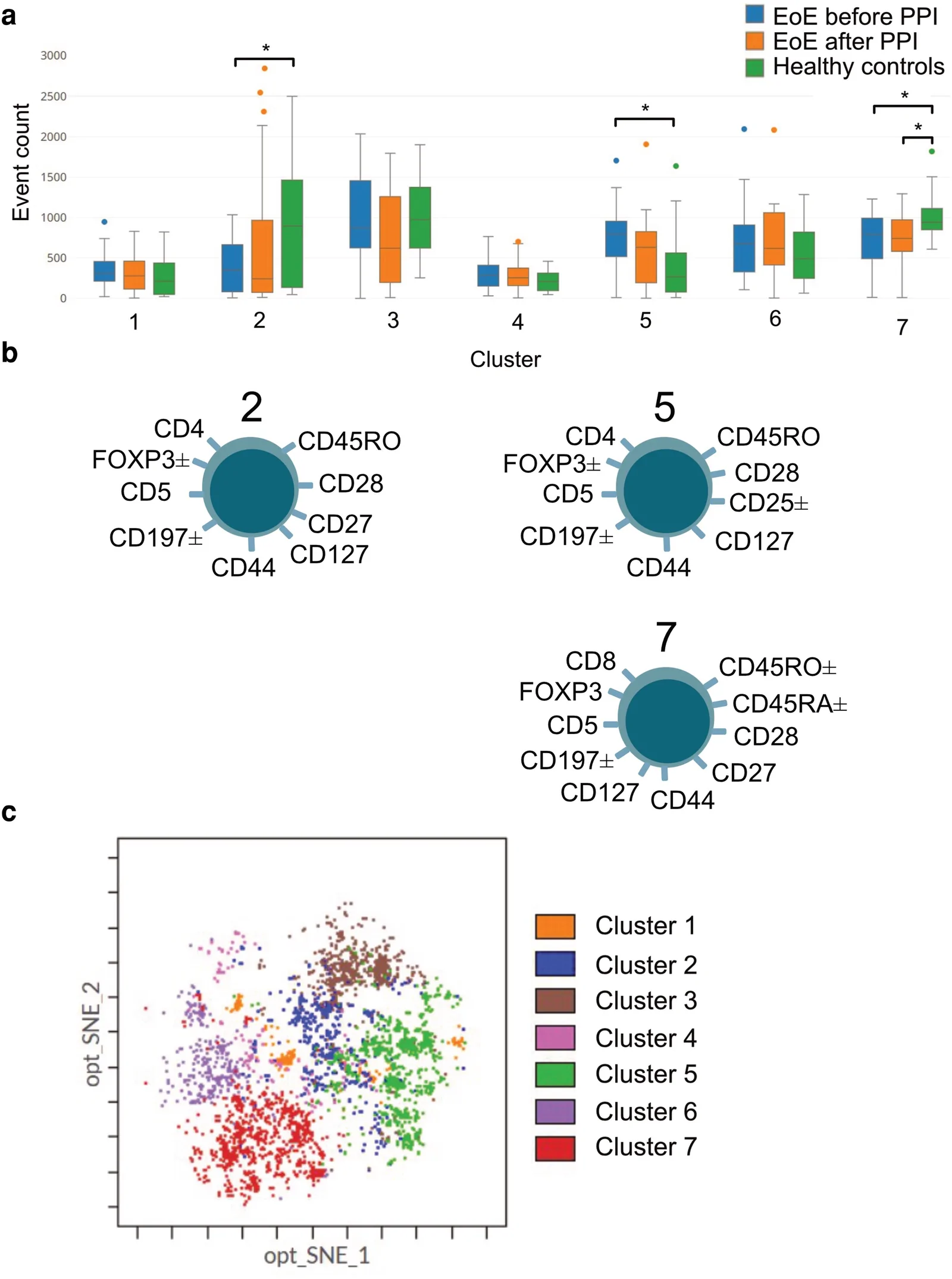
**(**a**)** Distribution of event counts across the seven T-cell clusters identified by FlowSOM in EoE patients before PPI treatment (blue, *n* = 17), after PPI treatment (orange, *n* = 17), and in healthy controls (green, *n* = 17). Boxplots show the full data range (min–max). Statistical comparisons between pre- and post-treatment samples were performed using the Wilcoxon paired test, while comparisons with healthy controls were assessed using the Mann–Whitney *U* test. **(**b**)** Marker expression profiles illustrating the phenotypes of the clusters of interest. **(**c**)** opt-SNE visualization map displaying the spatial distribution of the seven identified clusters in two-dimensional space. Event counts refer to the number of CyTOF events assigned to a given cluster following FlowSOM clustering and normalization. Median intensity reflects normalized marker expression levels within the identified population. *P*-values: one star (*): *P* < 0.05 and two stars (**): *P* < 0.01 were considered statistically significant.

To identify T-cell populations and visualize clustering differences after PPI treatment, and to compare these findings with age- and sex-matched controls, we performed unsupervised FlowSOM clustering on the CyTOF data. The FlowSOM algorithm identified seven distinct clusters based on marker expression profiles. Of these, three clusters were of particular interest: clusters 2, 5, and 7 (Fig. 2a). Based on their marker expression profiles, cluster 2 exhibited a phenotype consistent with Th2 central memory CD4^+^ T cells, whereas cluster 5 exhibited a phenotype consistent with FOXP3-expressing CD4^+^ T-cell populations and cluster 7 corresponded to a CD8^+^ T-cell population with naïve phenotype (Fig. 2b). Interestingly, cluster 2 was less abundant in EoE patients before treatment compared with healthy controls, whereas cluster 5 was more abundant in EoE patients at baseline than in controls. In addition, cluster 7 was more abundant in healthy controls compared with both the pre- and post-treatment samples from EoE patients. Although several comparisons did not remain significant after FDR correction, some exhibited moderate to large effect sizes, suggesting that limited statistical power due to sample size may have contributed to the lack of statistical significance, this additional data can be found in Supplementary Table S4. To further visualize the clusters, an opt-SNE map was generated using manually gated T cells from the CyTOF data, enabling visualization of the clusters in a two-dimensional scatterplot (Fig. 2c).

### T-cell phenotypic changes after proton pump inhibitor therapy and compared with healthy controls

**Figure 3:**
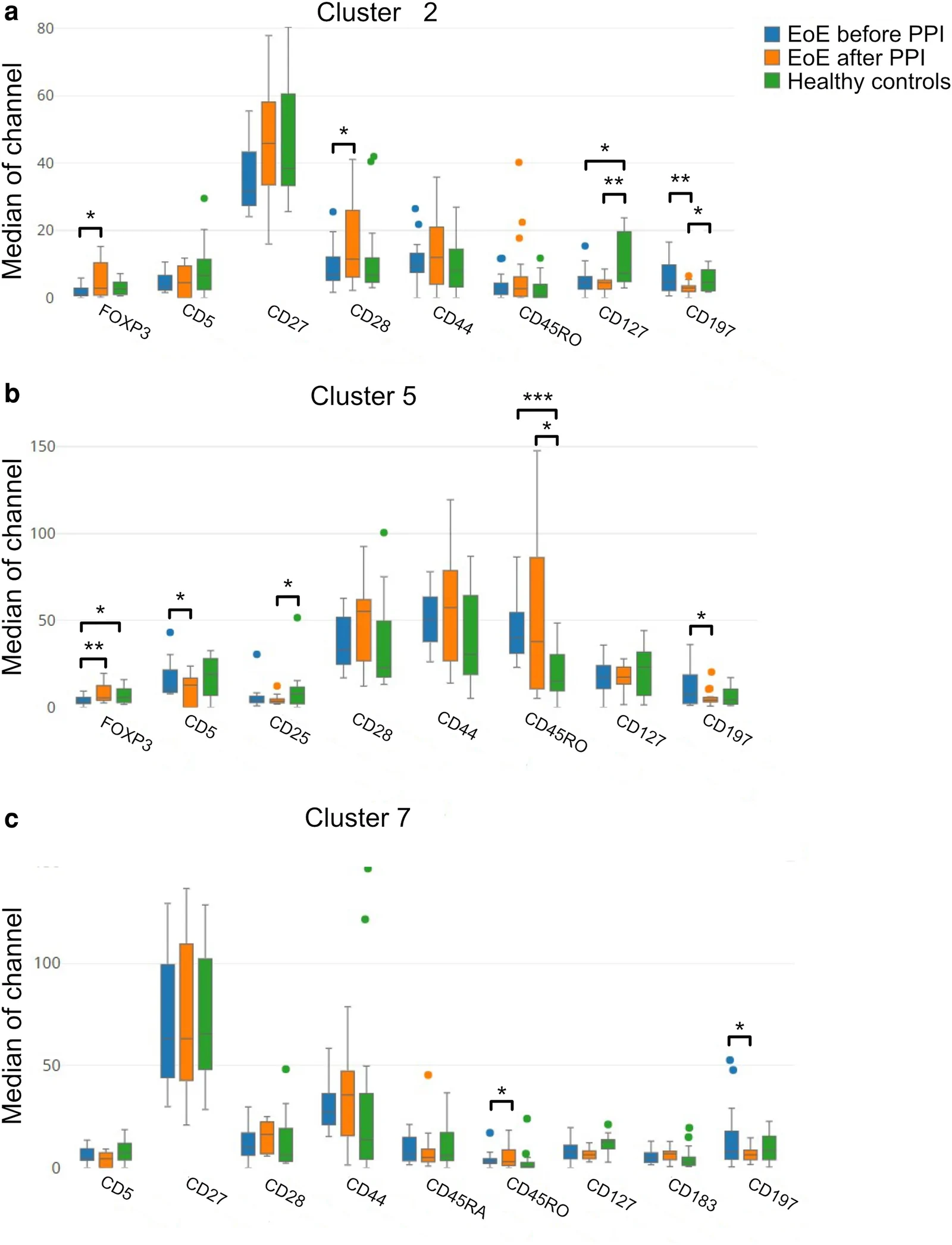
Median expression levels are shown for (a) Cluster 2), (b) Cluster 5, and (c) Cluster 7 in EoE patients before PPI (blue, *n* = 17) after PPI (orange, *n* = 17) and healthy controls (green ,*n* = 17). These clusters correspond to central memory-to-effector memory transitioning CD4+ T cells (Cluster 2)), Th2 central memory-to-regulatory-transitioning CD4+ T cells (Cluster 5) and naïve-to-effector-transitioning CD8+ T cells (Cluster 7)). Statistical comparisons were performed with the Wilcoxon paired test (pre- vs. post-treatment) and the Mann–Whitney *U* test (comparisons to healthy controls). Median intensity reflects normalized marker expression levels within the identified population. P-values: one star (*): *P* < 0.05 and two stars (**): *P* < 0.01 were considered statistically significant.

We next evaluated the clusters to characterize their marker expression profiles and assess statistical differences across groups. Cluster 2 demonstrated a phenotype consistent with Th2 central memory T cells based on expression of CD4+, CD197+, CD45RO+, CD185−, and CD183−. Following treatment, we observed decreased expression of CD197 (CCR7), a chemokine receptor involved in lymphocyte homing to secondary lymphoid tissues and commonly used to distinguish central memory from effector memory T-cell subsets. Reduced CCR7 expression may suggest a shift toward a more effector memory-like phenotype. In addition, expression of FOXP3 and CD28 increased following treatment, whereas CD127 levels remained higher in healthy controls than in both pre- and post-treatment EoE samples (Fig. 3a). FOXP3 is a transcription factor associated with regulatory T-cell differentiation and immune tolerance, CD28 is a co-stimulatory molecule involved in T-cell activation and survival and CD127, the IL-7 receptor α-chain, plays an important role in T-cell survival and homeostasis. Several marker differences exhibited moderate to large effect sizes despite not remaining significant following FDR correction, suggesting that limited statistical power may have contributed to the lack of statistical significance. Notably, CD127 expression remained significantly lower in EoE patients after treatment than in healthy controls (FDR-adjusted *P* = 0.036, Hedges’ *g* = 1.17), indicating a large effect size (Supplementary Table S4). Cluster 5 displayed a phenotype consistent with Th2 central memory T cells in EoE patients, whereas in healthy controls the same cluster also expressed CD25, indicating a regulatory T-cell phenotype. Consistent with this observation, FOXP3 expression was higher in healthy controls than in EoE patients before treatment. Interestingly, FOXP3 expression increased after treatment, reaching levels comparable to those of healthy controls. Treatment also resulted in reduced expression of both CD197 (CCR7) and CD5 (Fig. 3b). In addition, CD45RO expression in cluster 5 was higher in EoE patients before PPI treatment than in healthy controls. This difference remained significant following correction for multiple testing (Supplementary Table S4), indicating a robust effect. Cluster 7 consisted of naïve CD8^+^ T cells characterized by expression of CD197 and CD45RA before treatment but shifted toward an effector memory CD8^+^ T-cell phenotype after treatment, marked by increased CD45RO expression and loss of CD197 (Fig. 3c).

### FOXP3^+^ T-cell populations are associated with successful proton pump inhibitor therapy in EoE

**Figure 4:**
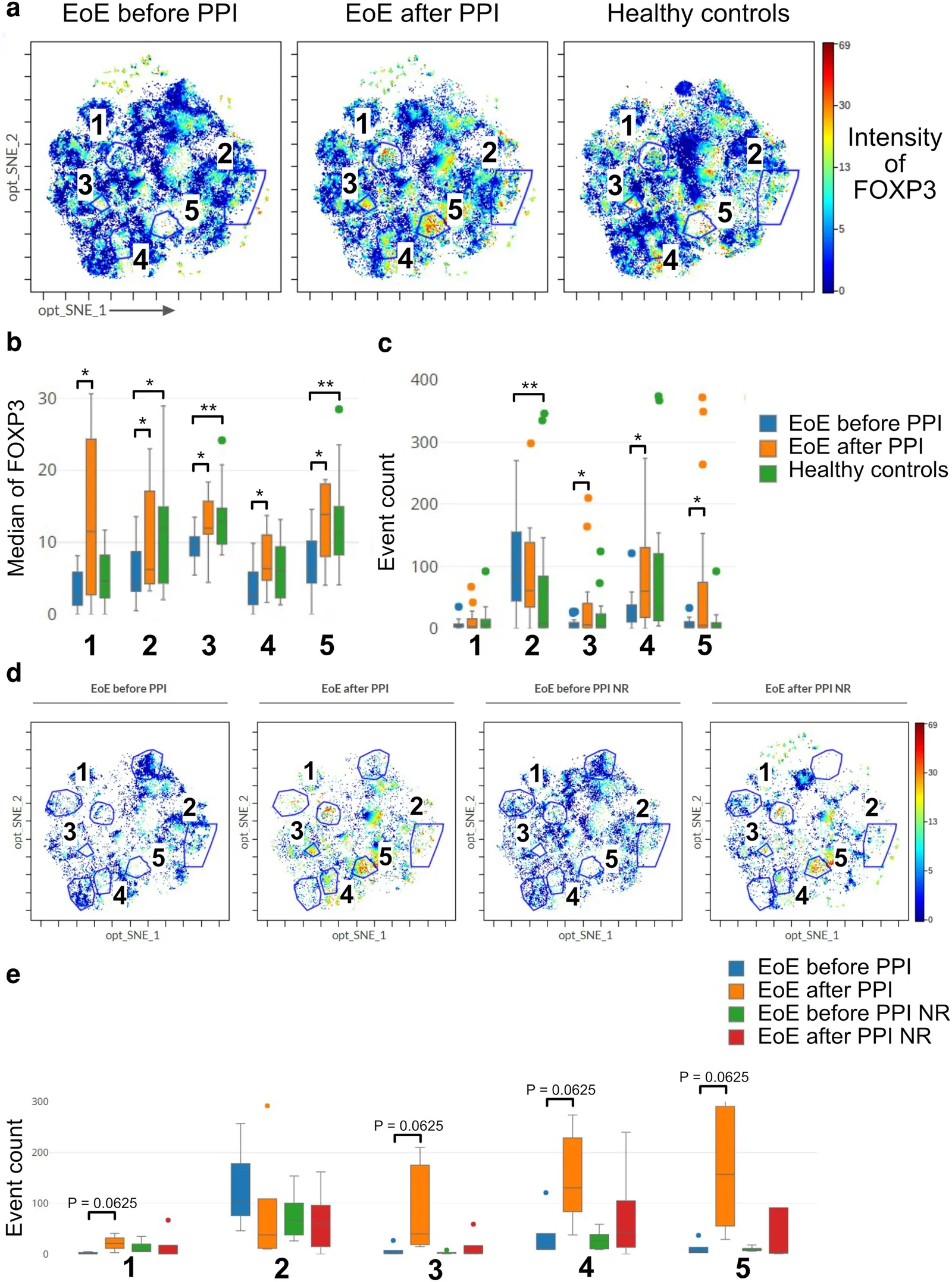
Opt-SNE map of distinct populations displaying shifts in cluster intensity in the treated group (*n* = 17). (a**)** FOXP3 intensity, (b**)** FOXP3 boxplots of median intensity, (c**)** FOXP3 boxplots of event counts (d**)** Opt-SNE map of distinct populations displaying shifts in cluster intensity in the successfully treated group (*n* = 5) which appeared following treatment, were notably less abundant or entirely absent in the non-responder group (*n* = 5). Five age- and sex-matched responders were selected for comparison with the five patients who did not respond. Each dot on the map represents a single event. Event counts refer to the number of CyTOF events assigned to a given cluster following FlowSOM clustering and normalization. Median intensity reflects normalized marker expression levels within the identified population. *P*-values: one star (*): *P* < 0.05 and two stars (**): *P* < 0.01 were considered statistically significant.

Next, we used the opt-SNE visualization map on manually gated T cells in the CyTOF data to identify differences in patterns between the three groups. FOXP3 expression increased notably in Populations 1–5, with higher median intensity after treatment in EoE patients (Fig. 4b). In addition, Populations 3–5 had displayed higher event count after treatment in EoE patients (Fig. 4c). Additionally, FOXP3 median intensity in Populations 2, 3, and 5 was higher in healthy controls compared with EoE patients before treatment (Fig. 4b), although differences in event count did not reach statistical significance (Fig. 4c). Calculations for FDR correction in presented in Supplementary Table S4.

Next, we conducted a comparative analysis between the five nonresponders to five responders. Nonresponders were defined as patients who remained above the remission threshold of <15 eosinophils per HPF following treatment. Five age- and sex-matched responders were selected for comparison with the five patients who did not respond (Fig. 4d). This analysis revealed that several FOXP3^+^ T-cell populations that appeared following treatment were notably less abundant or entirely absent in the nonresponder group (Fig. 4e).

### Reduction of CD56-expressing CD8^+^ T-cell populations following successful proton pump inhibitor therapy

**Figure 5:**
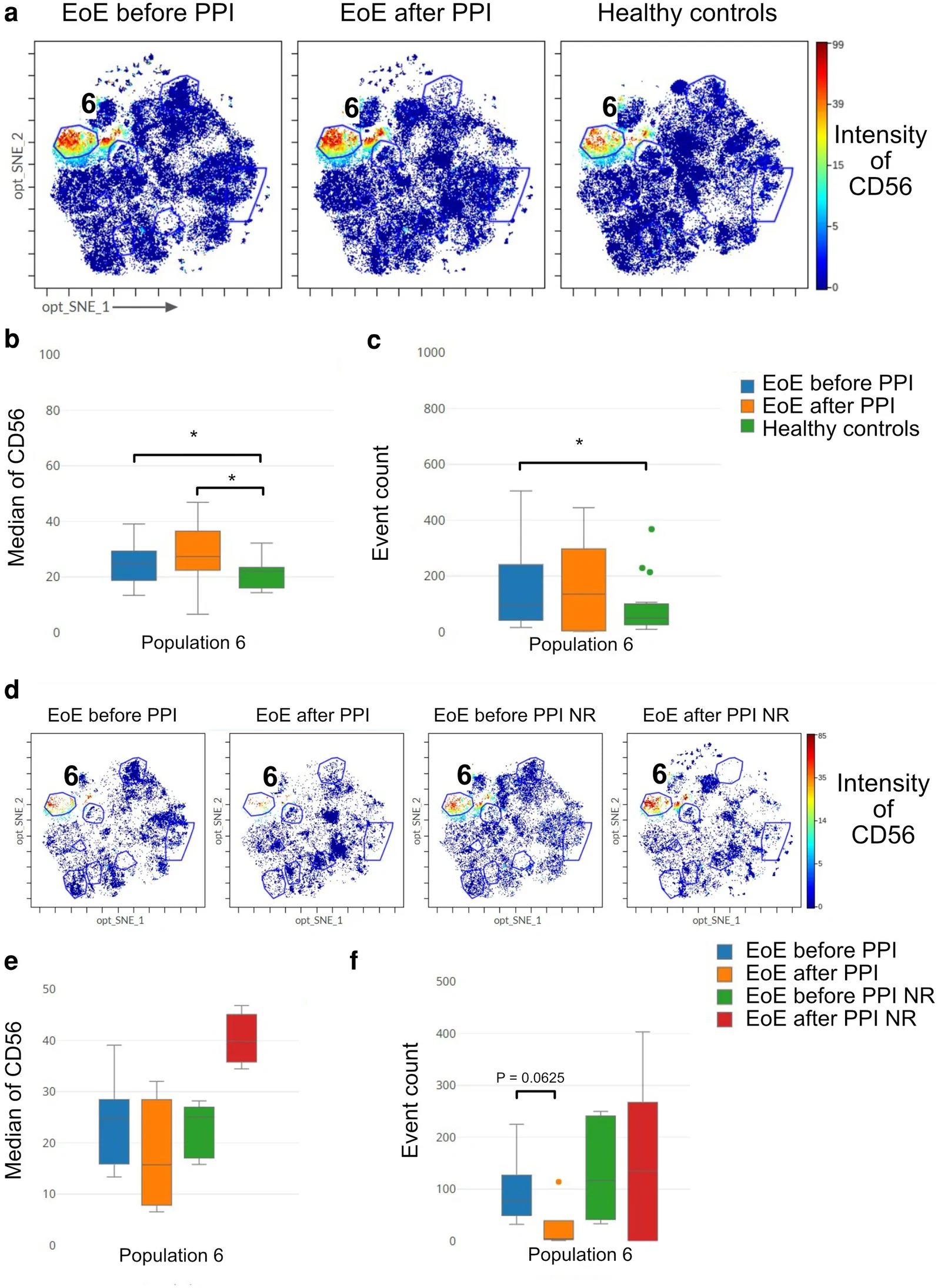
Opt-SNE map of distinct populations displaying shifts in cluster intensity in the treated group (*n* = 17). (a**)** CD56 intensity, (b) CD56 boxplots of median intensity, (c) CD56 boxplots of event counts (d) Opt-SNE map of distinct populations displaying shifts in cluster intensity in the successfully treated group (*n* = 5), CD56^+^ T-cell populations persisted exclusively in the non-responder group (*n* = 5). Five age- and sex-matched responders were selected for comparison with the five patients who did not respond. Each dot on the map represents a single event. Event counts refer to the number of CyTOF events assigned to a given cluster following FlowSOM clustering and normalization. Median intensity reflects normalized marker expression levels within the identified population. *P*-values: one star (*): *P* < 0.05 and two stars (**): *P* < 0.01 were considered statistically significant.

Subsequently, we used the opt-SNE visualization map to investigate differences in CD56 expression among the three groups (Fig. 5a). CD56 expression on T cells has been associated with activated or natural killer (NK)-like phenotypes. Notably, a CD8^+^ population exhibited markedly higher CD56 expression (Fig. 5b) and increased cell numbers (Fig. 5c) in EoE patients compared with healthy controls at both the pre- and post-treatment time points (Population 6). Calculations for FDR correction is presented in Supplementary Table S4.

When comparing the five nonresponders with five age- and sex-matched responders, we observed that the CD56^+^ T-cell populations persisted predominantly in the nonresponder group (Fig. 5d). Moreover, there was a clear trend toward increased CD56 expression following unsuccessful treatment (Fig. 5e). In contrast, successful treatment was associated with a near-significant reduction in CD56^+^ event counts (Fig. 5f).

### Treatment-associated reductions in naïve CD4^+^ and CD8^+^ T-cell populations

**Figure 6:**
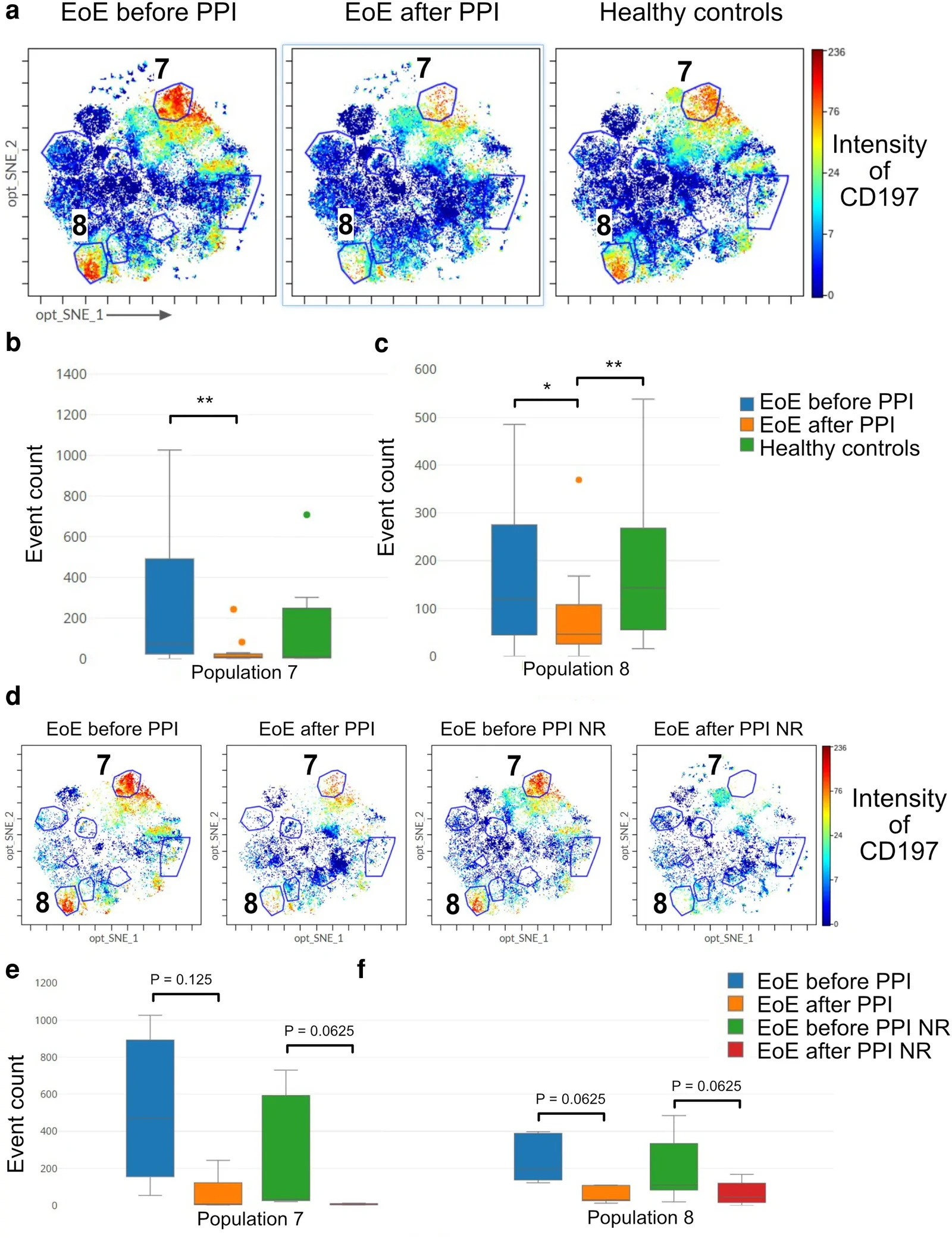
Opt-SNE map of distinct populations displaying shifts in cluster intensity in the treated group (*n* = 17). (a**)** CD197 intensity, (b**)** CD197 boxplots of event counts for population 7, (c) CD197 boxplots of event counts for population 8 (d) Opt-SNE map of distinct populations displaying shifts in cluster intensity in both responders (*n* = 5) and non-responders (*n* = 5). Five age- and sex-matched responders were selected for comparison with the five patients who did not respond. Each dot on the map represents a single event. Event counts refer to the number of CyTOF events assigned to a given cluster following FlowSOM clustering and normalization. Median intensity reflects normalized marker expression levels within the identified population. *P*-values: one star (*): *P* < 0.05 and two stars (**): *P* < 0.01 were considered statistically significant.

Next, we applied the opt-SNE visualization map to examine differences in naïve T-cell populations, defined by co-expression of CD197 and CD45RA, across the three groups (Fig. 6a). This analysis revealed notable shifts in naïve subsets following treatment. Specifically, a naïve CD4^+^ population (Population 7) showed a clear reduction in event counts after treatment compared with baseline levels in EoE patients (Fig. 6b). Similarly, a naïve CD8^+^ population (Population 8) displayed decreased event counts after treatment relative to both pretreatment samples and healthy controls (Fig. 6c), suggesting that naïve CD8^+^ T cells may be particularly sensitive to therapeutic modulation. Because similar reductions were observed in both responders and nonresponders, these findings likely represent treatment-associated immune changes rather than response-specific alterations. Calculations for FDR correction in presented in Supplementary table S4.

To further explore these observations, we compared five non-responders with five age- and sex-matched responders (Fig. 6d). Interestingly, both naïve CD4^+^ and naïve CD8^+^ populations showed a consistent trend toward reduced event counts in both responders and nonresponders following treatment (Fig. 6e–f). Although the magnitude of reduction varied between individuals, the overall pattern suggests a general contraction of naïve T-cell compartments in EoE patients after initiation of therapy, independent of clinical outcome.

### Histological findings in the esophagus correlate with circulating CD4^+^ T cell numbers

**Figure 7:**
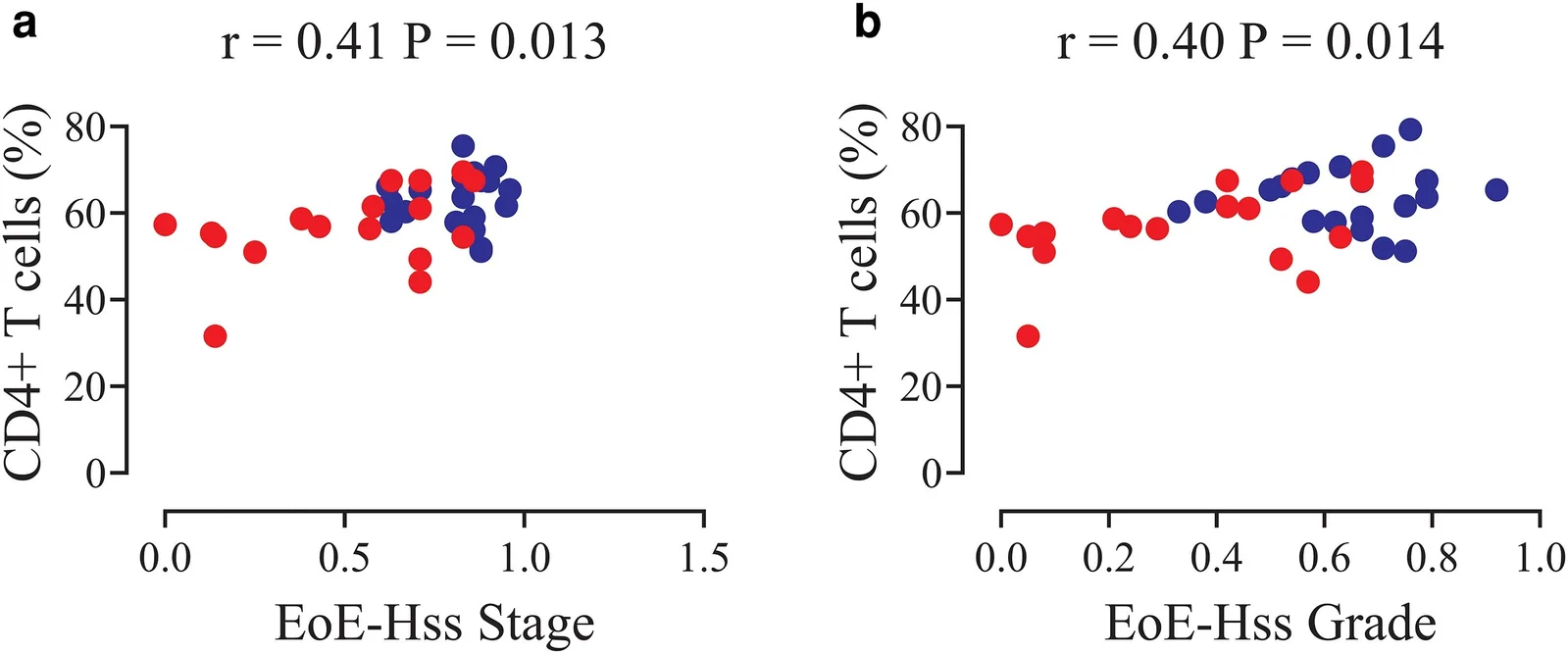
Univariate analysis of correlation between (a) EoE-HSS stage and CD4^+^ T cells and (b) EoE-HSS grade and CD4^+^ T cells. The red circles are EoE patients before treatment with proton pump inhibitors and the blue circles are EoE patients after treatment. *P*-values: *P* < 0.05 were considered statistically significant.

Finally, we explored whether any peripheral immune markers were associated with histologic disease severity, as measured by the EoE-HSS stage and grade, or with the number of eosinophils present in esophageal tissue. Identifying such correlations could provide insight into potential blood-based biomarkers capable of reflecting mucosal inflammation and treatment response in EoE. To address this, we performed a univariate correlation analysis across all measured T-cell populations and clinical parameters. Interestingly, both EoE-HSS stage and grade demonstrated a significant positive correlation with the number of circulating CD4^+^ T cells (Fig. 7a and b), suggesting that peripheral CD4^+^ T-cell levels may partially reflect tissue-level disease activity. In contrast, none of the examined T-cell subsets, including naïve, effector, memory, or regulatory populations, showed any meaningful correlation with eosinophil density (eosinophils/mm^2^) in esophageal tissue. This lack of association highlights an important dissociation between peripheral immune signatures and local eosinophilic infiltration, underscoring the complexity of using blood-based markers to predict tissue eosinophilia in EoE. However, peripheral immune changes may reflect systemic immune state, treatment exposure, or immune-cell redistribution, and not necessarily reflect local esophageal inflammation.

## Discussion

EoE is a chronic, immune-mediated esophageal disease characterized by eosinophil-predominant inflammation and symptoms of esophageal dysfunction. The immunological mechanisms underlying responsiveness and resistance to PPI therapy remain incompletely understood. In this study, using high-dimensional cytometry and unsupervised clustering approaches, we provide a detailed characterization of peripheral blood T-cell phenotypes in EoE patients before and after PPI treatment, compared with healthy controls. Our findings highlight significant shifts in T-cell subsets, particularly involving Th2 central memory, regulatory, cytotoxic, and naïve T-cell populations, which correlate with clinical and histologic disease markers.

In our cohort, PPIs effectively reduced esophageal eosinophil counts in 14 of 19 patients, confirming their clinical utility in EoE management. Immunophenotyping analyses were performed in 17 patients, comprising 12 responders and 5 nonresponders, because 2 responder samples contained insufficient cell numbers for reliable CyTOF analysis. The significant decrease in eosinophils after treatment corroborates previous findings [30, 31]. This histologic improvement was paralleled by reductions in EoE-HSS stage and grade, endoscopic findings (EREFS), and symptom scores (EEsAI and WDS), reflecting broad improvement of mucosal inflammation and clinical burden.

Importantly, these clinical improvements coincided with distinct immunophenotypic shifts. Our use of FlowSOM and opt-SNE clustering of CyTOF data revealed seven distinct T-cell clusters, with particular focus on Clusters 2, 5, and 7, corresponding to Th2 central memory CD4^+^ T cells, regulatory T cells, and naïve T cells, respectively. Notably, Cluster 2 was less abundant in EoE patients before treatment compared with controls but increased following PPI therapy, potentially reflecting restoration or expansion of this subset. Cluster 2 demonstrated a phenotype consistent with Th2 central memory T cells before treatment; however, after treatment, decreased expression of CD197 suggested a shift toward an effector memory T-cell phenotype. This observation is consistent with previous studies demonstrating decreased CD197 expression following treatment [32]. In addition, expression of FOXP3 and CD28 increased following treatment, whereas CD127 levels remained higher in healthy controls than in both pre- and post-treatment EoE samples. In addition, healthy controls exhibited low FOXP3 expression, suggesting that these cells may more closely resemble conventional CD4+ T cells rather than classical regulatory T cells. This finding is consistent with previous work reporting CD127^lo^ regulatory T cells in the esophagus of EoE patients, which they suggested could be memory effector Tregs [2]. CD127 plays an important role in T-cell survival and homeostasis. Persistently lower CD127 expression in patients with EoE may reflect altered T-cell activation states or immune dysregulation that is not fully normalized following treatment. However, the biological significance of this finding remains unclear and warrants further investigation.

Th2 responses have long been implicated in EoE pathogenesis, driving eosinophil recruitment through IL-5 and IL-13 production [1, 2]. The observed decrease in CD197 expression and concomitant increase in FOXP3 within cluster 2 after treatment suggest a phenotypic shift from central memory toward an effector memory or regulatory phenotype. FOXP3, a transcription factor characteristic of regulatory T cells (Tregs), was lower in EoE patients before treatment compared with healthy controls but increased after treatment, reaching levels comparable to those observed in controls. Interestingly, this effect was not observed in non-responders, further highlighting the immunological distinction between treatment outcomes. Previous studies have reported conflicting findings regarding Treg involvement in EoE. Some studies suggest a relative deficiency of Tregs in adult EoE that does not normalize after corticosteroid therapy [9] whereas others have demonstrated increased Treg numbers in EoE patients [33]. Our data align more closely with the former interpretation. However, it is also possible that Tregs migrate from peripheral blood into the inflamed esophageal mucosa during active disease, thereby reducing their detectable numbers in circulation.

Cluster 5 demonstrated a phenotype consistent with Th2 central memory T cells in patients before treatment but exhibited a regulatory T-cell phenotype after treatment and in healthy controls, marked by CD25 and FOXP3 expression. This finding further supports a reduction in circulating Tregs during active EoE followed by an increase after treatment. Moreover, the reduction in CD197 (CCR7) and CD5 expression after treatment indicates altered homing and activation states of these T cells, which could influence their migration to inflamed esophageal tissue. Furthermore, our results indicate a reduction in Th2-associated populations following PPI therapy, consistent with previous findings demonstrating that PPI treatment significantly downregulates esophageal eotaxin-3 and Th2-cytokine gene expression [34].

The CD8^+^ T-cell compartment (Population 6) displayed notable differences in CD56 expression. CD56, typically associated with NK cells and a subset of cytotoxic T cells, was elevated in untreated EoE patients compared with controls. This finding suggests that CD56^+^ CD8^+^ T cells may contribute to active esophageal inflammation, consistent with previous reports demonstrating elevated levels of cytotoxic lymphocytes in the esophageal mucosa of patients with active EoE [2]. The persistence of CD56^+^ T-cell populations in nonresponders, together with the trend toward increased CD56 expression following unsuccessful therapy, may indicate an association with treatment response. These findings identify CD56^+^ T-cell populations as candidate treatment-associated immune signatures that warrant further investigation in larger cohorts.

Cluster 7 demonstrated a phenotype consistent with naïve CD8^+^ T cells, showed a shift toward an effector memory phenotype after treatment, marked by loss of CD197 and increased expression of CD45RO, suggesting immune activation and differentiation. CD5 expression also decreased after therapy, which may reflect activation and differentiation into effector cells. The reduction of naïve CD4^+^ and CD8^+^ T-cell populations after treatment observed in both responders and nonresponders, indicates a generalized contraction of the naïve T-cell compartment following therapy. This pattern appears independent of clinical outcome, suggesting that the effect may be related to PPI treatment itself rather than disease-specific pathophysiology.

A central challenge in EoE management is the identification of noninvasive biomarkers that reflect esophageal inflammation. Our univariate analysis demonstrated that circulating CD4^+^ T-cell numbers correlated positively with histologic disease severity (EoE-HSS stage and grade), whereas none of the examined T-cell subsets, including naïve, effector, memory, or regulatory populations, showed any correlation with eosinophil density (eosinophils/mm^2^) in esophageal tissue. This lack of association highlights an important dissociation between peripheral immune signatures and local eosinophilic infiltration, underscoring the complexity of using blood-based markers to predict tissue eosinophilia in EoE. The lack of correlation between eosinophils and eosinophils/HPF may be explained by the patchy distribution of eosinophilic infiltration within the mucosa, where dense infiltration can occur in localized areas and may therefore be missed in biopsy samples. Although, even if circulating CD4^+^ T-cell numbers correlated with EoE-HSS stage and grade, many of the identified T-cell populations did not correlate with tissue eosinophil density. These findings suggest that peripheral immune signatures should carefully be interpreted as direct surrogates of local esophageal inflammation. Rather, the observed alterations in circulating immune populations may reflect broader systemic immune processes, treatment exposure, or redistribution of immune cells between the circulation and affected tissues.

The observed cellular alterations may reflect several pathophysiological mechanisms involved in EoE. Reduced FOXP3^+^ T-cell populations in active disease may indicate impaired immune regulation and insufficient suppression of Th2-driven inflammation, whereas the expansion of FOXP3 ^+^ CD25^+^ populations after successful PPI treatment suggests partial restoration of regulatory immune pathways. In contrast, the persistent absence or low abundance of these populations in non-responders may reflect ongoing immune dysregulation despite therapy. Persistently reduced CD127 expression in EoE patients compared with healthy controls further supports the presence of altered T-cell homeostasis and regulatory dysfunction. Alterations in CD197 (CCR7), CD5, and CD28 expression may indicate changes in T-cell activation, differentiation, and trafficking between blood and lymphoid tissues during treatment response. In addition, elevated CD56 expression in terminal effector CD8^+^ T cells suggests enrichment of cytotoxic or activated lymphocyte populations that may contribute to epithelial injury and chronic inflammation, particularly in non-responders where these populations persisted after treatment. The observed reduction of CD56^+^ populations following successful treatment may therefore reflect attenuation of inflammatory effector responses. Furthermore, shifts from naïve toward effector memory T-cell phenotypes may indicate ongoing immune remodeling during treatment. Finally, the positive correlation between circulating CD4^+^ T-cell populations and EoE-HSS severity scores suggests that systemic immune activation may partially reflect disease activity, although the lack of correlation with tissue eosinophil counts highlights the complexity of EoE pathogenesis and indicates that local eosinophilic inflammation may not be fully mirrored by peripheral blood immune signatures.

Our results advance the understanding of the immunological effects of PPI therapy in EoE. Beyond acid suppression, the mechanisms underlying the observed immune changes remain uncertain. The alterations in circulating T-cell populations observed in this study may reflect reduced disease activity, altered antigen exposure, immune-cell redistribution, or treatment-associated effects. The expansion of FOXP3^+^ T-cell populations following successful PPI therapy represents a key finding. These cells increased in responders but were notably reduced or absent in non-responders, raising the possibility that restoration of FOXP3-expressing T-cell populations may be associated with remission in EoE. Furthermore, the reduction in CD56^+^ CD8^+^ T cells highlights potential mechanisms associated with reduced inflammatory activity following treatment, possibly by dampening cytotoxic effector responses that may be associated with inflammatory processes contributing to epithelial injury.

Some limitations should be acknowledged. The modest sample size, particularly in responder versus nonresponder subgroup analyses, limiting statistical power and increasing susceptibility to interindividual variation. False discovery rate correction using the Benjamini–Hochberg procedure was applied where appropriate, and both raw and adjusted *P*-values are reported in the Supplementary material. In addition, while peripheral blood provides accessible insight into systemic immunity, it may not fully capture the local esophageal microenvironment. Integration of mucosal immunophenotyping and functional assays would provide a more comprehensive understanding of disease mechanisms. Peripheral immune alterations observed in this study may reflect systemic immune activation, treatment exposure, or redistribution of immune cells between blood and esophageal tissue rather than directly mirroring local esophageal inflammation. The absence of paired mucosal immunophenotyping precludes assessment of whether circulating immune changes correspond to alterations within the esophageal microenvironment. Future studies integrating paired blood and tissue analyses will therefore be essential.

## Conclusion

In summary, this study identifies distinct T-cell phenotypic changes following PPI therapy in EoE, including expansion of FOXP3^+^ T-cell populations and contraction of CD56-expressing CD8^+^ T-cell populations. Peripheral CD4^+^ T-cell counts correlated with disease severity, suggesting that circulating immune populations may reflect aspects of systemic disease activity. However, these findings should be considered exploratory and require validation in larger independent cohorts. These findings contribute to improved immunological understanding of EoE and may support future development of biomarker-driven and personalized therapeutic strategies.

## Supplementary Material

uxag051_Supplementary_Data

## Data Availability

The data supporting the findings of this study are available from the corresponding author upon reasonable request.

## Permission to reproduce

No previously published figures, tables, or other copyrighted material have been reproduced in this manuscript; therefore, permission to reproduce was not required.

## Clinical trial registration

Not applicable. This observational study was not a clinical trial and therefore was not registered in a clinical trial registry.

## Abbreviations:

CI: confidence interval
CyTOF: cytometry by time of flight
EEsAI: Eosinophilic Esophagitis activity index
EoE: eosinophilic esophagitis
EoE-HSS: eosinophilic esophagitis histology scoring system
EREFS: eosinophilic esophagitis endoscopic reference score
FDR: false discovery rate
HPF: high-power field
IL: interleukin
NK: natural killer
opt-SNE: optimized t-distributed stochastic neighbor embedding
PBS: phosphate-buffered saline
PPI: proton pump inhibitor
RT: room temperature
Th2: T helper 2
WDS: Watson dysphagia scale
