# Supplementary material for: Peripheral T-cell transitions following proton pump inhibitor therapy in eosinophilic esophagitis
Source: Clin Exp Immunol. 2026 Aug 20;220(1):uxag051. doi: 10.1093/cei/uxag051 (PMC13541109; doi:10.1093/cei/uxag051)
Supplement: Supplementary file 1 [file uxag051_supplementary_data.zip › Supplementary Material M&M for publication.docx]

**Supplementary table S3: Antibodies used for CyTOF**

| **Antibody** | **Clone** | **Metal label** | **Source** |
| --- | --- | --- | --- |
| CD45 | HI30 | 89Y | Fluidigm |
| IgG | G18-145 | 106Cd | In house |
| RANKL | #685857 | 110Cd | In house |
| CD69* | FN50 | 111Cd | In house |
| CD31* | WM59 | 112Cd | In house |
| CD1a | HI149 | 113Cd | In house |
| CD5* | UCHT2 | 114Cd | In house |
| IgA | HP6123 | 116Cd | In house |
| CD196/CCR6* | G034E3 | 141Pr | Fluidigm |
| CD19 | HIB19 | 142Nd | Fluidigm |
| CD127/IL-7Ra* | A019D5 | 143Nd | Fluidigm |
| CD38* | HIT2 | 144Nd | Fluidigm |
| IL-5R | 26815 | 145Nd | In house |
| IgD | IA6-2 | 146Nd | Fluidigm |
| CD8 | RPA-T8 | 146Nd | Fluidigm |
| CD11c | Bu15 | 147Sm | Fluidigm |
| CD274* | 29E.2A3 | 148Nd | Fluidigm |
| CD34* | 581 | 149Sm | Fluidigm |
| CD44* | IM7 | 150Nd | Fluidigm |
| CD123/IL-3R | 6H6 | 151Eu | Fluidigm |
| TCRgd* | 11F2 | 152Sm | Fluidigm |
| CD185/CXCR5* | RF8B2 | 153Eu | Fluidigm |
| CD3 | UCHT1 | 154Sm | Fluidigm |
| CD45RA* | HI100 | 155Gd | Fluidigm |
| CD14 | HCD14 | 156Gd | Fluidigm |
| CD27* | L128 | 158Gd | Fluidigm |
| FOXP3* | 259D/C7 | 159Tb | Fluidigm |
| CD28* | CD28.2 | 160Gd | Fluidigm |
| CD294/CRTH2 | BM16 | 161Gd | In house |
| CD66b | 80H3 | 162Dy | Fluidigm |
| CD183/CXCR3* | G025H7 | 163Dy | Fluidigm |
| Siglec-8 | 7C9 | 164Dy | Fluidigm |
| CD45RO* | UCHL1 | 165Ho | Fluidigm |
| CD24* | ML5 | 166Er | Fluidigm |
| CD197/CCR7* | G043H7 | 167Er | Fluidigm |
| CD8* | SK1 | 168Er | Fluidigm |
| CD199 | L053E8 | 168Er | Fluidigm |
| CD25 | 2A3 | 169Tm | Fluidigm |
| Galectin-10 | B-F42 | 170Er | In house |
| CD20 | 2H7 | 171Yb | Fluidigm |
| IgM | MHM-88 | 172Yb | Fluidigm |
| HLA-DR* | L243 | 173Yb | Fluidigm |
| CD4* | SK3 | 174Yb | Fluidigm |
| CD193/CCR3* | 5E8 | 175Lu | Fluidigm |
| CD56* | NCAM16.2 | 176Yb | Fluidigm |
| DNA1 | - | 191Ir | Fluidigm |
| DNA2 | - | 193Ir | Fluidigm |
| Cell-ID Cisplatin | - | 195Pt | Fluidigm |
| CD16 | 3G8 | 209Bi | Fluidigm |

*used for clustering in FlowSOM and opt-SNE analysis

**Supplementary table S4**

| **EoE before PPI n = 17**  **EoE after PPI n = 17**  **Healthy controls n = 17** | **P-value** | **Benjamini–Hochberg FDR correction** | **Bonferroni correction** | **Mean difference** | **95% confidence interval** | **Effect sizes (Hedges´g)** |
| --- | --- | --- | --- | --- | --- | --- |
| Figure 2 |  |  |  |  |  |  |
| Cluster 2 - EoE before PPI vs healthy controls | **0.020** | **0.034** | 0.74 | 12.32 | 3.22 to 21.41 | 0.94 |
| Cluster 5 - EoE before PPI vs healthy controls | **0.013** | **0.031** | 0.48 | −8.04 | −14.64 to −1.43 | −0.83 |
| Cluster 7 - EoE before PPI vs healthy controls | **0.034** | **0.042** | 1 | 6.25 | 1.58 to 10.92 | 0.91 |
| Cluster 7 - EoE after PPI vs healthy controls | **0.045** | **0.046** | 1 | 5.53 | 0.86–10.20 | 0.81 |
| Figure 3 |  |  |  |  |  |  |
| Cluster 2 |  |  |  |  |  |  |
| FOXP3 - EoE before PPI vs EoE after PPI | **0.015** | **0.031** | 0.56 | 3.71 | 0.95–6.47 | 0.91 |
| CD28 - EoE before PPI vs EoE after PPI | **0.015** | **0.031** | 0.56 | 7.19 | 0.02 to 14.36 | 0.69 |
| CD127 - EoE before PPI vs healthy controls | **0.018** | **0.033** | 0.67 | −5.88 | −10.02 to −1.74 | −0.96 |
| CD127 - EoE after PPI vs healthy controls | **0.0030** | **0.022** | 0.11 | 6.99 | 3.14 to 10.85 | 1.17 |
| CD197 - EoE before PPI vs EoE after PPI | **0.0021** | **0.022** | 0.078 | −3.27 | −5.82 to −0.72 | −0.90 |
| CD197 - EoE after PPI vs healthy controls | **0.0475** | **0.048** | 1 | 2.41 | 0.65 to 4.17 | 0.94 |
| Cluster 5 |  |  |  |  |  |  |
| FOXP3 - EoE before PPI vs EoE after PPI | **0.0026** | **0.022** | 0.096 | 4.58 | 1.58–7.58 | 1.07 |
| FOXP3 - EoE before PPI vs healthy controls | **0.012** | **0.031** | 0.44 | 0.13 | 0.02–0.24 | 0.84 |
| CD5 - EoE before PPI vs EoE after PPI | **0.027** | **0.039** | 0.99 | −118.5 | −352 to 115 | −0.36 |
| CD25 - EoE after PPI vs healthy controls | **0.027** | **0.039** | 0.99 | 1.25 | 0.17–2.33 | 0.78 |
| CD45RO - EoE before PPI vs healthy controls | **0.00056** | **0.021** | **0.021** | 22.9 | 11.6–34.1 | 1.38 |
| CD45RO - EoE after PPI vs healthy controls | **0.031** | **0.041** | 1 | 33.66 | 10.3–57.0 | 1.00 |
| CD197 - EoE before PPI vs EoE after PPI | **0.0057** | **0.026** | 0.21 | 1.52 | 0.53–2.51 | 1.04 |
| Cluster 7 |  |  |  |  |  |  |
| CD45RO - EoE before PPI vs EoE after PPI | **0.0086** | **0.030** | 0.32 | 2.73 | −1.21 to 6.67 | 0.47 |
| CD197 - EoE before PPI vs EoE after PPI | **0.0076** | **0.029** | 0.28 | 90.5 | −104 to 285 | 0.32 |
| Figure 4B |  |  |  |  |  |  |
| Population 1 - EoE before PPI vs EoE after PPI | **0.0034** | **0.022** | 0.13 | 9.78 | 3.45–16.11 | 1.22 |
| Population 2 - EoE before PPI vs EoE after PPI | **0.044** | **0.046** | 1 | 4.61 | 0.5 to 8.72 | 0.79 |
| Population 2 - EoE before PPI vs healthy controls | **0.022** | **0.034** | 0.81 | 6.50 | 1.91–11.09 | 1.06 |
| Population 3 - EoE before PPI vs EoE after PPI | **0.022** | **0.034** | 0.81 | 3.21 | 0.93 to 5.49 | 0.98 |
| Population 3 - EoE before PPI vs healthy controls | **0.016** | **0.033** | 0.59 | 4.04 | 1.16 to 6.92 | 1.09 |
| Population 4 - EoE before PPI vs EoE after PPI | **0.011** | **0.031** | 0.41 | 3.94 | 1.53 to 6.35 | 1.15 |
| Population 5 - EoE before PPI vs EoE after PPI | **0.011** | **0.031** | 0.41 | 6.09 | 2.52 to 9.66 | 1.24 |
| Population 5 - EoE before PPI vs healthy controls | **0.015** | **0.031** | 0.56 | 5.96 | 1.61 to 10.31 | 1.05 |
| Figure 4C |  |  |  |  |  |  |
| Population 2 - EoE before PPI vs healthy controls | **0.022** | **0.034** | 0.81 | 33.3 | −31.1 to 97.7 | 0.35 |
| Population 3 - EoE before PPI vs EoE after PPI | **0.036** | **0.042** | 1 | 27.11 | -2.7 to 56.9 | 0.62 |
| Population 4 - EoE before PPI vs EoE after PPI | **0.036** | **0.042** | 1 | 56.59 | 12.7 to 100.5 | 0.90 |
| Population 5 - EoE before PPI vs EoE after PPI | **0.036** | **0.042** | 1 | 66.17 | 4.4 to 128.0 | 0.73 |
| Figure 5B |  |  |  |  |  |  |
| Population 6 - EoE before PPI vs healthy controls | **0.015** | **0.031** | 0.56 | 5.71 | 1.36 to 10.06 | 0.95 |
| Population 6 - EoE after PPI vs healthy controls | **0.037** | **0.042** | 1 | 6.87 | 0.82 to 12.92 | 0.82 |
| Figure 5C |  |  |  |  |  |  |
| Population 6 - EoE before PPI vs healthy controls | **0.033** | **0.042** | 1 | 99.37 | 22.7 to 176.0 | 0.89 |
| Figure 6B |  |  |  |  |  |  |
| Population 7 - EoE before PPI vs EoE after PPI | **0.0037** | **0.022** | 0.14 | 235.96 | 60.1 to 411.8 | 0.94 |
| Figure 6C |  |  |  |  |  |  |
| Population 8 - EoE before PPI vs EoE after PPI | **0.042** | **0.046** | 1 | 97.37 | 18.7 to 176.1 | 0.86 |
| Population 8 - EoE after PPI vs healthy controls | **0.01** | **0.031** | 0.37 | 118.17 | 41.7 to 194.7 | 1.08 |

Confidence intervals and effect sizes were calculated from group means and standard deviations and are provided as descriptive measures. Mean differences were calculated as the second group minus the first group listed in each comparison. Statistical significance was assessed using non-parametric tests as described in Materials and Methods. P-values (p < 0.05) are indicated in bold. Adjusted p-values are based on 37 statistical comparisons.

**Supplementary figure S1.**

**Supplementary figure S1.** Gating strategy for samples analyzed with CyTOF. The gating is based on intact, single, live cells. Followed by gating of granulocytes from lymphocytes, dendritic cells, monocytes and NK cells. Eosinophils are gated by using CD294 and Siglec-8 from the granulocyte gate. The expression of the eosinophil markers not used in the eosinophil gating were evaluated for each sample. Gating of CD4+ T cells are depicted on the second and third row showing activated, central memory (CM), naïve, effector memory (EM), and, terminal effector (TE) CD4+ T cells, the same procedure was done for CD8+ T cells. Tregs are gated based on the expression of CD25 and CD127. Gating of Th1, Th2 and Th17 cells are shown on the fourth row and B cells on the fifth row. Rows six and seven are demonstrating the gating of plasmablast, monocytes, NK cells and dendritic cells, both myeloid (mDC) and plasmacytoid (pDC). This gating strategy has previously been published elsewhere (1).

References

1.	Larsson H, Albinsson Hogberg S, Lind M, Rabe H, Lingblom C. Investigating immune profile by CyTOF in individuals with long-standing type 1 diabetes. Sci Rep. 2023;13(1):8171.
