# Supplementary material for: Peripheral T-cell transitions following proton pump inhibitor therapy in eosinophilic esophagitis
Source: Clin Exp Immunol. 2026 Aug 20;220(1):uxag051. doi: 10.1093/cei/uxag051 (PMC13541109; doi:10.1093/cei/uxag051)
Supplement: Supplementary file 1 [file uxag051_supplementary_data.zip › Supplementary Material Patients for publication.docx]

# **Supplementary Tables**

| **Patient ID** | **Age** | **Sex** | **Type of allergy** | **Other conditions** | **Eos/HPF**  **(size HPF mm^2^)** | | **Eos/mm^2^** | | **EREFS (0-8)** | | **EoE-Hss Grade (0-1)** | | **EoE-Hss**  **Stage (0-1)** | | **EEsAi Pro-score (0-100)** | | **Watson Dysphagia Score (0-45)** | |
| --- | --- | --- | --- | --- | --- | --- | --- | --- | --- | --- | --- | --- | --- | --- | --- | --- | --- | --- |
|  |  |  |  |  | **Before** | **After** | **Before** | **After** | **Before** | **After** | **Before** | **After** | **Before** | **After** | **Before** | **After** | **Before** | **After** |
| HP01^b^ | 70 | M |  |  | 24 (0.19) | 1 (0.19) | 126 | 5 | 5 | 1 | 0.5 | 0.12 | 0.71 | 0.21 | 34 | 0 | 14 | 4 |
| HP02^b^ | 48 | M |  |  | 22(0.21) | 0 | 105 | 0 | 4 | 0 | 0.63 | 0.42 | 0.92 | 0.63 | 36 | 0 | 19 | 18.5 |
| HP03^b, c, i^ | 34 | M | Pollen, milk, wheat, peanuts |  | 59(0.21) | 0 | 281 | 0 | 2 | 2 | 0.92 | 0.42 | 0.96 | 0.58 | 60 | 27 | 25.5 | 19 |
| HP04^b,h^ | 19 | M |  |  | 16(0.19) | 35(0.21) | 84 | 167 | 4 | 3 | 0.75 | 0.63 | 0.95 | 0.83 | 27 | 0 | 8 | 0 |
| HP05^b^ | 63 | M |  | ENT ca^e^ | 42(0.21) | 0 | 200 | 0 | 6 | 1 | 0.58 | 0.24 | 0.63 | 0.43 | 67 | 61 | 22.5 | 19 |
| HP06^b^ | 47 | M | Pollen, mite |  | 91(0.21) | 5(0.21) | 433 | 24 | 3 | 2 | 0.75 | 0.08 | 0.88 | 0.25 | 34 | 0 | 13.5 | 0 |
| HP07 | 38 | F | Not tested |  | 26(0.21) | 1(0.21) | 124 | 5 | 0 | 0 | 0.38 | 0.08 | 0.63 | 0.13 | 74 | 59 | 22.5 | 23 |
| HP08^h^ | 34 | M | Not tested |  | 26(0.19) | 83(0.21) | 137 | 395 | 4 | 3 | 0.71 | 0.67 | 0.83 | 0.83 | 57 | 27 | 18 | 14.5 |
| HP09^a^ | 42 | M | Not tested |  | 17(0.21) | X | 81 | X | 5 | X | 0.76 | X | 0.81 | X | 63 | X | 16 | X |
| HP10^b^ | 55 | M |  |  | 16(0.19) | 9(0.19) | 84 | 47 |  | 3 | 0.57 | 0.46 | 0.86 | 0.71 | 34 | 27 | 21.5 | 10 |
| HP11^b^ | 45 | M | Pollen, mite, fish |  | 37(0.21) | 0 | 176 | 0 | 3 | 3 | 0.52 | 0.19 | 0.62 | 0.33 | 61 | 27 | 17.5 | 11 |
| HP12^i^ | 30 | M | Fur |  | 21(0.19) | 9(0.19) | 111 | 47 | 2 | 3 | 0.62 | 0.57 | 0.81 | 0.71 | 46 | 12 | 12 | 15 |
| HP13^b^ | 57 | M | Mite, fur |  | 24(0.21) | 0 | 114 | 0 | 1 | 3 | 0.33 | 0 | 0.67 | 0 | 63 | 55 | 23 | 22.5 |
| HP14^b, i^ | 75 | M | Pollen. mite. fur | Asthma, BPH^f^ | 75(0.21) | 0 | 357 | 0 | 5 | 3 | 0.67 | 0.21 | 0.86 | 0.38 | 0 | 12 | 8 | 0 |
| HP15^b^ | 57 | F | Pollen. Fur | RA^g^ | 108(0.21) | 0 | 514 | 0 | 3 | 3 | 0.67 | 0.29 | 0.86 | 0.57 | 72 | 63 | 17.5 | 22.5 |
| HP16^b, d, i^ | 49 | M |  |  | 38(0.21) | 0 | 181 | 0 | 5 | 1 | 0.71 | 0.05 | 0.88 | 0.14 | 12 | 12 | 11 | 7.5 |
| HP17^b, d, i^ | 33 | M | Pollen, |  | 16(0.19) | 0 | 84 | 0 | 2 | 2 | 0.54 | 0.05 | 0.83 | 0.14 | 52 | 46 | 21 | 11.5 |
| HP18^b, d, h^ | 76 | F |  |  | 72(0.21) | 24(0.21) | 343 | 114 | 5 | 0 | 0.67 | 0.67 | 0.9 | 0.86 | 15 | 15 | 12 | 12 |
| HP19^b, d, h^ | 51 | M | Pollen, fur, |  | 122(0.21) | 20(0.19) | 581 | 105 | 7 | 4 | 0.79 | 0.54 | 0.88 | 0.71 | 0 | 12 | 4.5 | 4.5 |
| HP20^b, h^ | 29 | M | Pollen, fur |  | 41(0.19) | 65(0.21) | 216 | 310 | 4 | 1 | 0.79 | 0.52 | 0.83 | 0.71 | 61 | 0 | 15 | 0 |

**Supplementary table 1.** Demographics and clinical features of EoE patients

a) excluded from the study, b) RAST test c), skin prick test, d) no food allergens tested e) ear nose throat cancer, f) previous benign prostate hypertrophy, g) reumatoid arthritis, h) non-responder, i) age- and sex- matched responder for non-responders/responders analysis

**Supplementary table 2.** Characteristics of EoE patients

|  | **Before treatment**  **N = 19** | **After treatment**  **N = 19** |
| --- | --- | --- |
| Eosinophils/HPF  (0.19 - 0.21mm^2^) | 45 (16 - 122) | 13 (0 - 83) |
| EoE-Hss Grade | 0.64 (0.33 – 0.92) | 0.33 (0 – 0.67) |
| EoE-Hss Stage | 0.82 (0.62 – 0.96) | 0.48 (0 – 0.86) |
| EREFS | 3.7 (0 - 7) | 2 (0 - 4) |
| Watson Dysphagia Score | 16.1 (4.5 – 25.5) | 11.3 (0 - 23) |
| EEsAI | 43.4 (0 - 74) | 24 (0 - 63) |

Numbers are mean value with min/max
